# Pain Characteristics and Quality of Life in Older People at High Risk of Future Hospitalization

**DOI:** 10.3390/ijerph18030958

**Published:** 2021-01-22

**Authors:** Maria M Johansson, Marco Barbero, Anneli Peolsson, Deborah Falla, Corrado Cescon, Anna Folli, Huan-Ji Dong

**Affiliations:** 1 Unit of Clinical Medicine, Division of Prevention, Rehabilitation and Community Medicine, Department of Acute Internal Medicine and Geriatrics and Department of Health, Medicine and Caring Sciences, Linköping University, SE-581 83 Linköping, Sweden; 2 Rehabilitation Research Laboratory 2rLab, Department of Business Economics, Health and Social Care, University of Applied Sciences and Arts of Southern Switzerland, CH-6928 Manno/Landquart, Switzerland; marco.barbero@supsi.ch (M.B.); corrado.cescon@supsi.ch (C.C.); anna.folli@supsi.ch (A.F.); 3 Unit of Physiotherapy, Division of Prevention, Rehabilitation and Community Medicine, Department of Health, Medicine and Caring Sciences, Linköping University, SE-581 83 Linköping, Sweden; anneli.peolsson@liu.se; 4 Centre of Precision Rehabilitation for Spinal Pain (CPR Spine), School of Sport, Exercise and Rehabilitation Sciences, College of Life and Environmental Sciences, University of Birmingham, Birmingham B15 2TT, UK; d.falla@bham.ac.uk; 5 Pain and Rehabilitation Centre, and Department of Health, Medicine and Caring Sciences, Linköping University, SE-581 83 Linköping, Sweden; huanji.dong@liu.se

**Keywords:** aging, quality of life, vulnerable, pain extent, pain drawing, primary care

## Abstract

This study deals with how pain characteristics in conjunction with other factors affect quality of life (QoL) in a vulnerable primary care population. We recruited vulnerable older people (75+, *n* = 825) living in south-eastern Sweden. A postal questionnaire included pain aspects, QoL (EQ-5D-3L, RAND-36 physical functioning, attitudes toward own aging, and life satisfaction), functional status, social networks, and basic demographic information. Pain extent and localization was obtained by digitalization of pain drawings reported on standard body charts. Most respondents were experiencing pain longer than 3 months (88.8%). Pain frequency varied mostly between occasionally (33.8%) and every day (34.8%). A minority reported high pain intensity (13.6%). The lower back and lower legs were the most frequently reported pain locations (>25%). Multiple linear regression model revealed three characteristics of pain (intensity, frequency, and extent) remained inversely associated with the EQ-5D-3L index score (R^2^ = 0.57). Individually, each of these pain characteristics showed a negative impact on the other three dimensions of QoL (R^2^ = 0.23–0.59). Different features of pain had impact on different dimensions of QoL in this aging population. A global pain assessment is useful to facilitate individual treatment and rehabilitation strategies in primary care.

## 1. Introduction

Pain is highly prevalent among older people. It is suggested that up to 93% of older people have some degree of pain [1]. This varies depending on the study population, methods applied, and pain definition used. The economic burden of pain for society and the health care system is unquestionably substantial [2,3]. In an aging population, a greater extent of pain is associated with several comorbidities and medication use [4], suggesting a potentially high cost of healthcare consumption, ultimately due to hospitalization. As the aging population continues to increase, it is reasonable to expect that the number of hospitalizations and health care spending for older adults will rise. A good understanding of prevention, pain management, and rehabilitation within primary care is therefore of great importance. The high prevalence of pain among elderly may lead to a false assumption that pain is normal in old age [5,6]. However, besides the awareness of pain existence, it is relevant to consider how the extent of pain impacts on disability and poor health [7]. Most studies sought to determine the extent of pain in older people by considering a general aging population [8,9,10], whereas only a few studies have examined pain characteristics among vulnerable or hospitalized patients (65 and over) [11,12]. Additionally, previous studies that evaluated vulnerable people over 80 years, described common pain locations but did not quantify the extent of pain (size of the painful area) [8,10,11]. To address the knowledge gap of pain characteristics in vulnerable older adults in primary care, this study investigates pain characteristics of people with a predicted high-risk for future hospitalization [13,14]. Since pain can be a significant cause of suffering and significantly affects quality of life (QoL) [15,16], we also wanted to examine the impact of pain on multi-dimensional aspects of QoL to provide knowledge and indications for targeted group-specific interventions in order to maintain, restore, or improve QoL of vulnerable older people. QoL and functional ability should be the first focus of the multi-professional team in primary care as well as in community care. Thus, the present study is also aimed at identifying which characteristics of pain are associated with different dimensions of QoL among the vulnerable older people. However, QoL covers different conceptualizations and there are no standard indicators of QoL. To select the relevant indicators with a focus on functional ability, we followed a biopsychosocial framework of pain and its consequences on QoL, including biological, physical, psychological, and social aspects.

## 2. Materials and Methods

### 2.1. Study Population

A detailed process description of patient involvement was published as a study protocol [13]. Briefly, a prospective cohort design considered all residents aged 75 and over in the county of Östergötland (*n* = 40,728) located in the southeast of Sweden during the period April–December 2017. Östergötland is the fourth largest county in Sweden with 460,000 inhabitants in total and includes both urban and rural areas. From 2016 to 2030, the proportion of older people (≥65 years old) is estimated to increase by 16%, and the proportion of the population 85 years old and older is expected to increase by 40% [17]. A case-finding algorithm (prediction for hospital care) was used to identify eligible persons within this population [14]. The sample size for the required study was calculated (*n* = 1600) based on an unpublished pilot study that showed 60% of the target population had one or more hospital admissions during a 12-month period. A postal questionnaire with an invitation letter was sent to the high-risk population of 1487 persons still alive when the study started. In cases of reduced autonomy due to mental disease or other reasons, close relatives or caregivers could participate and provide help with filling out the questionnaire. 

### 2.2. Ethical Considerations

The study was approved by the regional ethical review board in Linköping (Dnr: 2016/347-31). 

### 2.3. Measures

Data presented in the current study consist of the baseline data collected in the longitudinal study. The information was combined with life-course data on the person and family from Statistics Sweden (SCB). Listed below are the variables and instruments used in this study. 

#### 2.3.1. Sociodemographic Factors

Age (years), gender, marital status, living situation (living alone, together with partner, or with children), housing (own housing, sheltered accommodation, or nursing home), highest education (less than nine-years’ of schooling, nine-years of schooling, secondary school, or university/college) were included. 

#### 2.3.2. Pain Characteristics

Pain frequency. The respondents were asked to assess their pain frequency, with response options: 1 = “never”; 2 = “occasionally”; 3 = “once per day”; 4 = “several times per day”; and 5 = “constantly”. This variable was denoted as a 5-likert scale.

Pain duration. How long have you been suffering from pain (months)? This variable was denoted as pain duration. According to the current version of the International Classification of Diseases (ICD-11) developed by the International Association for the Study of Pain (IASP), longer than three months was categorized as chronic pain [18].

Pain intensity. Pain intensity over the preceding seven days was measured using visual analogue scale (VAS Pain–7d, 0 = no pain and 100 = worst imaginable pain) [19]. Mild pain was defined with VAS pain scores of 30 mm or below, moderate pain with scores from 31 to 69 mm, and severe pain with scores of 70 mm or more.

Pain extent. Quantification of pain extent was obtained from the pain drawings [20,21]. Respondents were asked to draw their pain on two body charts, showing a frontal and dorsal view of the body. Subsequently, all pain drawings were digitalized using a commercial scanner and stored on a PC. Each scanned image was scaled and aligned to a predefined size (1024 × 78 pixel) and the pain drawing was processed to place it in a separate layer with respect to the background body chart. Pain drawings were then processed to count the total number of pixels of the pain drawing in the frontal and dorsal body charts. 

Topographic pain maps. The pain drawings were overlaid to generate topographic pain maps, with the purpose of illustrating the most frequent pain locations across the entire sample. Both dorsal and ventral views are presented using a color map to illustrate the percentage of the respondents experiencing pain for a given body region.

#### 2.3.3. Functional Status

Use of assistive technology (AT). AT included both mobility and personal care devices, such as a wheelchair, walker, crutch, bath/shower technology, and adapted toilet [22]. Respondents could also choose “some others” if they used some other type of AT. Glasses, contact lenses, and hearing aids are extremely common devices in this age strata, so they were not counted. This variable was denoted as no use of AT/use any type of AT. The number of used AT for each respondent was also counted.

Physical activity. Respondents were asked about everyday physical activity and physical exercise during the last 6 months (see Supplemental Material). A four-point score (1 = inactivity, 2 = low activity, 3 = moderate activity, and 4 = high activity) was generated based on the combination of the results from the two questions [23].

Activities of daily living (ADL). ADL-staircase is an extended version of Katz’ ADL index that contains 10 activities including feeding, continence, transferring, going to the toilet, dressing, bathing, cooking, shopping, cleaning, and transportation [24]. Each activity has a three-graded scale of dependency (1 = independent, 2 = partly dependent, 3 = dependent). A total sum from 10–30 is calculated with a higher score indicating greater dependency. Good construct validity has been reported previously [25] and good internal consistency (Cronbach’s alpha of 0.89) was demonstrated in the present study.

#### 2.3.4. Social Networks

Having relatives nearby. Do you have relatives who live nearby? (having relatives nearby; only having relatives in another city/county, not having relatives). This variable was dichotomized into having relatives nearby vs. not.

Number of close relationships. How many persons in your life do you have close relationship with? This variable was denoted as number of close relationships.

Loneliness and worries. Participants were asked about feelings of loneliness and worries, both with the same response options (1 = yes, very often, 2 = sometimes, 3 = seldom, and 4 = never). The two variables were denoted as 4-likert scales.

#### 2.3.5. Quality of Life (QoL)

EQ-5D-3L. The EQ-5D-3L is a generic instrument that assesses health-related QoL (HRQoL) in terms of mobility, self-care, usual activities, pain/discomfort, and anxiety/depression [26,27]. Response alternatives are no problems, moderate problems, or extreme problems. Using the five dimensions, the instrument calculates an index (EQ5D-index) [27]. The reliability coefficient of the index in this study resulted in a Cronbach’s α of 0.71, which is an acceptable level of internal consistency. 

RAND-36 physical functioning (RAND-36_PF_). RAND-36 is one of the generic profile HRQoL measures [28]. A subscale of the RAND-36 with 10 items was used to determine physical functioning (RAND-36_PF_); higher scores (range: 0–100) indicated better physical functioning. A high reliability (Cronbach’s α of 0.93) was estimated in the present study.

Attitudes about own aging (ATOA). Lawton defined ATOA as the “ability to strive appropriately, while still accepting the inevitable”. ATOA was measured by the subscale of the Philadelphia Geriatric Center Morale Scale (PGCMS-subscale) [29]. The subscale included five dichotomous items (yes/no) that captured the individual’s perception of the changes in his or her life and a subjective evaluation of those changes. According to the scoring instructions, each answer indicating positive ATOA was scored with one point and answers indicating negative ATOA were scored zero. A sum of scores ranged from 0 to 5, with higher scores indicating more positive ATOA. The Swedish version of PGCMS showed acceptable validity and feasibility among older people [30]. The reliability (Cronbach’s alpha of 0.63) in this study was low but acceptable.

Life satisfaction. Satisfaction with life scale (SWLS) is a global measure of satisfaction with life as an overall summation of a person’s life satisfaction [31]. We adopted a 5-point scale (1 = strongly disagree−5 = strongly agree) and a sum of total score ranging from 5 to 25 [31]. Higher scores indicated greater satisfaction with life. Reliability (Cronbach’s alpha of 0.84) was satisfactory.

### 2.4. Statistical Analysis

Analyses were performed using the statistical package IBM SPSS Statistics (version 26.0; IBM Inc., New York, NY, USA). All data are reported as mean and standard deviation (SD), median with interquartile range (IQR), or number with percentage based on the distribution of data. The topographic pain maps and pain location maps were presented to provide a detailed description of the somatic distribution of pain across the whole study population. Multiple linear regression (MLR) analyses were performed to estimate the impact of pain characteristics on different QoL dimensions. Together with pain characteristics, other variables such as sociodemographic factors, functional status, and social network were included in the MLR models using a stepwise method. Both R^2^ and Akaike information criterion (AIC) were observed for model selections since the stepwise method could lead to model overfitting (overestimation of R^2^) [32,33]. In the current regression analysis, AIC values were consistently the lowest at the last step of the model building in all the four models. Multicollinearity was assessed by examining tolerance and the variance inflation factor (VIF). We further examined collinearity among the categorical variables using the phi (Φ, Φ ≥ 0.30 indicating high correlation) [34]. Two variables, marital status and living situation, showed high correlations. We selected living situation as it had fewer missing data points compared to marital status. Living alone was treated as a reference variable, whilst living with partners and living with children were recoded into two separate binary variables (dummy coding). Model fit was assessed by examining the distribution of the standardized residuals. Normality of the residuals was evaluated using the Shapiro–Wilk test in combination with visualized Q-Q plots.

MLR analyses use listwise deletion to handle the missing cases. In case missing data could potentially lead to biased results, we also performed multiple imputation to handle the missing data as a comparison to the results based on “real completed cases” (see Supplementary Material).

## 3. Results

In total, 853 out of 1487 persons answered the questionnaire (response rate of 57%). The current study population was restricted to all the respondents who replied to one or more questions on pain characteristics (*n* = 825, response rate 55.5%). A flow chart was used to describe the participation, inclusion, and exclusion of this study (see Supplementary Material). About one-fifth of the study population (*n* = 179, 21.7%) received the help of others, of which 36 (4.4%) cases were reported only by their relatives or caregivers. 

### 3.1. Participant Characteristics: Social Demographics, Functional Status, and Social Network

Sample characteristics are shown in Table 1. The overall mean age was 84.2 ± 5.6 years with almost equal representation of both genders. Nearly half of the respondents were living together with someone. Nine out of ten lived in their own house or apartment. A minority of this sample earned university or college education (16.6%).

Two-thirds of the respondents reported that they used at least one AT (median 1, IQR 0–3). The low median score of ADL-staircase suggested low ADL dependence. Physical activity level varied greatly among the respondents. Few were categorized as being highly physically active (7.1%) or with low activity (18.3%), and an approximately equal number of respondents were inactive or moderately active.

Most respondents in this study population had relatives living nearby (75.5%) and had close relationship with other people (99.5%). Almost every other respondent (48.2%) could count at least six important persons in their lives. However, one-third of all the respondents sometimes or often felt lonely and nearly 50% reported they sometimes or often felt worries about the future.

### 3.2. Pain Characteristics

Nearly equal proportions of the respondents had pain occasionally (33.8%) or daily (34.8%, Table 2). Just over one-tenth of the respondents (13.4%) reported constant pain, or severe pain (13.6%), and only a minority (9.7%) did not report pain. The median score of the VAS pain-7d was 40 mm (IQR 21–60), indicating moderate pain. Most respondents reported experiencing pain for more than 3 months (88.8%). The median pain extent was 1.93% with a limited variability in terms of size (IQR: 0.61–4.77).

#### Pain Location

**Figure 1:**
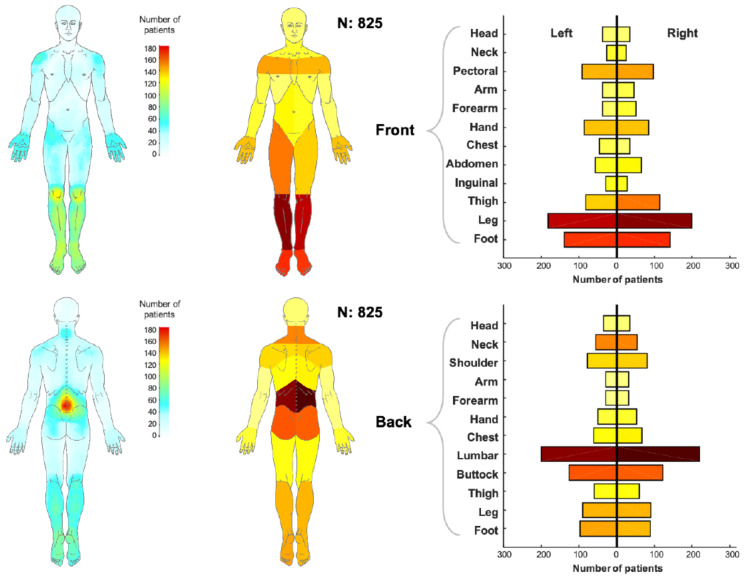
Topographic pain maps, on the left side, have been generated to highlight the most frequently reported pain locations for both the dorsal and ventral views. The color grid indicates the number of individuals that reported pain in that specific area. Red and yellow colors represent the most frequently reported areas of pain. Pain location maps, on the right side, indicate the number of persons who reported pain in a particular anatomical region. The regions of the body have been color coded as displayed on the left side of the column chart. The presence of the pain in a body region was confirmed when at least 10% of the body region area (corresponding to the number of pixels being greater than 60) was covered in the pain chart.

Figure 1 shows the topographical pain maps, generated by superposition of all the pain drawings of the 825 patients, to highlight the areas where the largest number of patients reported pain. In the frontal view, the knees and lower legs are in green/yellow color, indicating about 120 patients reported pain in those regions, hands and shoulders are in cyan color, indicating about 60 patients reported pain in those regions. In the back view, the lower back (and in particular the area around the lumbar vertebrae) is in red color indicating about 180 patients reported pain in that area. The same data are represented in the body charts on the right (and the corresponding histograms). In this case, the entire region (e.g., head, left hand, right thigh, etc.) is colored according to the number of patients reporting pain in that area. Over 25% of the respondents reported pain in the lower back, and about 20% of patients reported pain in knees and lower legs.

### 3.3. QoL and MLR Models

The median or mean values of QoL dimensions are listed in Table 1. The respondents rated their HRQoL generally high (median EQ-5D index 0.66) but with a low level of physical functioning (median score of RAND-36_PF_, 35). A low median score of PGCMS-subscale indicates negative attitude towards one’s own aging. However, the mean score of SWLS was higher than an average score, suggesting high satisfaction with life.

Table 3 summarizes the MLR analysis results by each dimension of QoL. Three characteristics of pain, VAS Pain-7d (β = −0.003, 95% confidence interval (CI) = −0.004, −0.003, *p* < 0.001), pain frequency (β = −0.05, 95% CI = −0.08, −0.02, *p* < 0.01), and pain extent (β = −0.005, 95% CI = −0.01, −0.001, *p* < 0.01), were negatively and significantly associated with the EQ-5D index. In the other three MLR models, only one pain characteristic showed a significant impact on each QoL parameter. These significant associations remained in the models when missing cases were handled using multiple imputations (see Supplemental Material).

Together with pain characteristics, several sociodemographic factors also contributed to the MLR models significantly. Moreover, all the functional status variables showed significant effects on RAND-36_PF_ and that physical activity level was positively related to the PGCMS-subscale and SWLS. Higher frequency of feeling lonely or worried for the future significantly decreased all the scores of QoL dimensions apart from the RAND-36_PF._

## 4. Discussion

This study presents an overview of pain characteristics in a vulnerable aging primary care population at high risk of future hospitalization. Using pain drawings to quantify pain location and extent, we identified a high prevalence of pain in the lower back, knees, and lower legs in this population. These findings suggested a high prevalence of underlying musculoskeletal disorders such as low back pain and osteoarthritis. Both specific (pathological changes such as muscle and joint strain, disc degeneration or prolapse, osteoarthritic and osteoporotic bone disease, and inflammatory spinal disease) and non-specific low back pain (the anatomic factors causing back pain do not fully explain existing pain symptoms) are common in older adults [35,36]. Similarly, the high prevalence of pain in the lower legs likely reflects the high prevalence of osteoarthritis in those over 80 [37]. According to Global Burden of Disease (GBD) estimates, these two disorders were among the most burdensome disorders for older adults [37]. Several characteristics of pain showed a negative impact on different dimensions of QoL. Our results are in accordance with the findings that pain in older people can have a negative influence on QoL in many ways, such as a general health disturbance, deterioration of physical functioning, negative attitude towards aging, and reduced life satisfaction [16,38,39].

Older people are expected to be a heterogenous group, and their pain characteristics were no exception. The descriptive data showed the variations of pain frequency, severity, and extent in the study population. This is the first study as far as we know to report pain extent in those over 80. Compared to other populations such as middle age populations with chronic low back pain, neck pain, or hip osteoarthritis, pain was relatively locally distributed with only a few individuals demonstrating widespread pain [20,40,41]. Although pain contributes to frailty onset and progression, it does not necessarily need to be constant, severe, or widespread pain. It should be noted that pain of a long-lasting duration (persistent or chronic pain) was particularly common in this vulnerable group at risk for hospitalization, with a much higher percentage compared to the general aging population [4,42]. This observation is supported by other studies that indicate that chronic pain is a common health issue for vulnerable older people at risk for multiple adverse health outcomes [9,43].

QoL is a broad, multi-dimensional concept. In this study, different instruments were used to measure QoL to enhance the understanding of QoL in this group and its associated factors. The EQ-5D index as an overall HRQoL was influenced by most pain features, underlying the important role of pain characteristics in HRQoL estimation. We also found that features of pain had different impacts on other specific QoL dimensions. In terms of physical functioning, pain intensity had the strongest impact. This finding was also reported in some other studies considering general aging populations [44,45]. Two other features of pain, namely pain extent and pain frequency, were negatively associated with psychological dimensions of QoL (attitude towards own aging and life satisfaction). This finding discourages the belief that pain is normal in old age [5,6]. Among patients with chronic pain, pain extent can be associated with the presence of psychological distress [21,40,46]. Collectively, these results reveal the necessity of a global assessment of pain in vulnerable older people. Further research is encouraged to explore the causal relationship between features of pain and QoL. Interestingly, pain duration did not affect any dimension of QoL in this population. One possible explanation is that chronic pain was extremely common (prevalence of 89%) in this special population. In the literature, among the frail patients aged 60 or older, the estimated prevalence of chronic pain was between 45–75% [9]. One early study on pain prevalence in the general Swedish population demonstrated that pain prevalence is likely to increase with the arrival of the 1940s generation to the oldest age groups [47].

Besides pain, sociodemographics, functional status, and social network showed impact on QoL. For example, advancing age was positively related to life satisfaction, in line with other studies [48,49]. This observation could be explained by wisdom increasing with age, which is defined as having “expert knowledge in the fundamental pragmatics of life”, the tendency towards reflection on one’s own behavior and that of others, and exhibiting kindness and empathy rather than egotism [50]. In addition, living situation and having relatives nearby were positively related to life satisfaction. Living alone as well as a bad family function are known to have a negative impact on QoL [51,52]. Expectedly, variables on functional status were significantly related to HRQoL of the physical functioning dimension (Rand-36_PF_). Psychosocial experience such as frequency of feeling lonely and worried contributed strongly to the psychological dimensions of QoL (PGCMS-subscale and SWLS). This was also found in other studies of older people with vulnerability or frailty [53,54]. This fact must be considered when planning for interventions in pain rehabilitation as well.

### Strengths and Limitations

To best of our knowledge, using a contemporary method, this is the first study to quantify the location and extent of pain in a population with a high proportion of older people aged 80 and over. One strength of the study was the pain drawing analysis, which provided topographic maps of pain distribution for the whole study sample. When analyzing the pain drawings to estimate pain extent and location, the software eliminated estimation errors (for example, the software is a deterministic system in which no randomness is involved) that potentially occur with visual-subjective scoring methods [20]. As applied in other pain studies, the software quantifies pain extent so that this feature can be analyzed statistically [21,55]. However, a limitation of this approach is the requirement of cognitive capacity; older people with severe impaired cognitive function might not be capable of providing a valid pain drawing. The bias should be considered as a minority of the questionnaire responses were from close relatives or caregivers due to reduced autonomy of our participants.

Our study explored the associations of different features of pain and different dimensions of QoL in older people over 75 at high-risk of future hospitalization. In comparison, previous studies considered general aging populations or hospitalized patients (65 and over) [8,9,10,11,12]. However, some other valuable information, such as specific diagnosis of pain-related diseases and medications, were not collected in this questionnaire. The study population was selected based on the predictive model that was generated by several predictors including 32 diagnostic codes of morbidities. These morbidities correspond to unplanned admission to hospital other than for pain-related diseases. Moreover, our cross-sectional analysis did not uncover any causation. This is planned for a future study. A follow-up study measuring changes of pain could validate the relationship observed from our data as well as investigate whether the intervention had any effects on pain and QoL. 

Response bias is a further possible limitation. Based on the registered data from SCB, there was no age difference between the non-respondents and respondents, but a slightly higher proportion of women than men (54.7% vs. 45.3%, *p* = 0.009) were among the non-respondents. Among the respondents, it seems that the percentage of people with pain was much higher than reported in studies on frail older people or hospitalized older adults [9,12]. The representation of this study population did not cover the older people with severe cognition impairment, since questions on pain evaluation required enough cognitive capacity to answer. We also suspect that respondents who completed all the questions on pain characteristics did so because they were more likely to have concerns about pain. To handle the excluded cases in listwise deletion and retain all available information from the respondents, we also performed a multiple imputation statistical approach (see Supplemental Material). The similar significance of regression estimates suggests a minimal influence of the missing values.

## 5. Conclusions

This study demonstrated that in this vulnerable older primary care population, chronic pain is common and most often located in the lower back, knees, and lower legs. Different features of pain may impact on different dimensions of QoL, when accounting for sociodemographic factors, functional status, and social network. For vulnerable older people, a global assessment of pain, rather than focusing only on pain intensity, is beneficial to gain a broad understanding of the influence of pain on QoL and is useful in tailoring both individual and group-specific treatment and rehabilitation. Future studies should focus on how the intervention has affected pain and QoL.

## Figures and Tables

**Table 1:** Sociodemographics, physical functions, social network, and quality of life.

	Total, *n* (%)	Range
Age (years), mean ± SD	84.2 ± 5.6	75–104
Gender, male, *n* (%)	430 (52.1)	
Marital status (*n* = 814), *n* (%)		
Married/co-habited	399 (49.2)	
Unmarried/widow/widower	412 (50.8)	
Living situation (*n* = 814), *n* (%)		
Living alone	413 (50.7)	
Living with partners	389 (47.2)	
Living with children	12 (1.5)	
Housing, *n* (%)		
Own house/apartment	750 (91.9)	
Sheltered accommodation/nursing home	66 (8.1)	
Education levels (*n* = 797), *n* (%)		
Less than 9 years of school	341 (42.8)	
9 years of school	79 (9.9)	
Secondary school	245 (30.7)	
University/college	132 (16.6)	
Use any type of assistive technology, *n* (%)	549 (68.5)	
Number of assistive technologies (*n* = 801), median (IQR)	1 (0–3)	0–6
ADL-staircase score (*n* = 807), median (IQR)	13 (11–17)	10–30
Physical activity (*n* = 579) ^a^, median (IQR)	2 (1–3)	1–4
Inactivity, *n* (%)	214 (37)	
Low activity, *n* (%)	106 (18.3)	
Moderate activity, *n* (%)	218 (37.7)	
High activity, *n* (%)	41 (7.1)	
Having relatives nearby (*n* = 803), *n* (%)	623 (75.5)	
Number of close relationships (*n* = 766), median (IQR)	6 (3–10)	0–67
None	4 (0.5)	
1–2	132 (16)	
3–5	232 (28.1)	
≥6	398 (48.2)	
Frequency of feeling lonely (*n* = 720) ^a^, median (IQR)	3 (2–4)	1–4
No, never/wish to be self, *n* (%)	280 (33.9)	
Yes, seldom, *n* (%)	168 (20.4)	
Yes, sometimes, *n* (%)	200 (24.2)	
Yes, often, *n* (%)	72 (8.7)	
Frequency of feeling worried (*n* = 725) ^a^	3 (2–4)	1–4
No, never, *n* (%)	183 (25.2)	
Yes, seldom, *n* (%), *n* (%)	196 (27)	
Yes, sometimes, *n* (%)	258 (35.6)	
Yes, often, *n* (%)	88 (12.1)	
EQ-5D index (*n* = 755), median (IQR)	0.66 (0.36–0.73)	−0.594–1
RAND-36 physical functioning (*n* = 802), median (IQR)	35 (15–60)	0–100
PGCMS-subscale (*n* = 745), median (IQR)	1 (0–2)	0–5
SWLS scale (*n* = 657), mean ± SD	16.8 ± 4.5	5–25

^a^ 4-likert scale. SD: standard deviation; IQR: interquartile range; ADL: activities of daily living; PF: physical functioning; PGCMS: Philadelphia Geriatric Center Morale Scale; SWLS: Satisfaction with life scale.

**Table 2:** Pain characteristics.

	Total, *n* (%)	Min–Max
Pain frequency (*n* = 782)		
Never	76 (9.7)	
Occasionally	264 (33.8)	
Everyday	272 (34.8)	
Several times per day	65 (8.3)	
Constant	105 (13.4)	
Pain duration (*n* = 562), months, median (IQR)	17 (9–36)	0–99
Less than three months	63 (11.2)	
Three months or longer	499 (88.8)	
Pain intensity (*n* = 704), VAS pain-7d, median (IQR)	40 (21–61)	0–100
0–30, none to mild pain	276 (33.5)	
31–69, moderate pain	316 (38.3)	
70–100, severe pain	112 (13.6)	
Pain extent (*n* = 825), percentage, median (IQR)	1.93 (0.61–4.77)	0–64.40

IQR: interquartile range; VAS: visual analog scale.

**Table 3:** Multiple linear regression models of quality of life.

DV		EQ-5D Index	RAND-36PF	PGCMS-Subscale	SWLS
IV					
VAS pain-7d		−0.003 (0.001) c	−0.19 (0.05) a	EXCL	EXCL
Pain frequency		−0.05(0.02) b	EXCL	EXCL	−0.59 (0.25) a
Pain extent		−0.005 (0.002) b	EXCL	−0.03 (0.01) b	EXCL
Age		EXCL	EXCL	EXCL	0.15 (0.05) b
Gender (reference: male)		EXCL	−5.71 (2.27) a	EXCL	EXCL
Education level		EXCL	EXCL	0.14 (0.06) a	EXCL
Living situation (reference: living alone)					
Living with partners		EXCL	EXCL	EXCL	1.62 (0.68) a
Living with children		EXCL	EXCL	EXCL	EXCL
Number of used assistive technologies		EXCL	−3.61 (1.11) b	EXCL	EXCL
Physical activity level		EXCL	8.83 (1.22) c	0.25 (0.07) c	1.16 (0.26) c
ADL-staircase		−0.03 (0.003) c	−2.01 (0.31) c	EXCL	EXCL
Having relatives nearby		EXCL	EXCL	EXCL	2.13 (0.62) b
Frequency of feeling lonely		−0.05 (0.01) b	EXCL	-0.20 (0.07) b	−0.72 (0.33) a
Frequency of feeling worried		−0.06 (0.01) c	EXCL	−0.33 (0.075) c	−0.85 (0.28) b
Constant		1.48 (0.06) c	70.15 (6.17) c	1.68 (0.30) c	4.90 (4.45)
R2		0.57	0.59	0.23	0.30

^a^*p* < 0.05, ^b^ *p* < 0.01, ^c^ *p* < 0.001. Coefficients (standard error). Excluded variables in all the models: pain duration, housing (own housing vs. nursing home), and number of close relationships. IV: independent variables; DV: dependent variables; EXCL: excluded from the model with stepwise selection; VAS: visual analog scale. PGCMS: Philadelphia Geriatric Center Morale Scale; SWLS: Satisfaction with life scale.

## Data Availability

Data are available from the corresponding author on reasonable request.

## Supplementary Materials

The following are available online at https://www.mdpi.com/1660-4601/18/3/958/s1, File 1; Physical activity measures and multiple imputation statistical method. File 2; Table S1. Physical activity level generated by two questions, Table S2. Missing cases in each variable, Table S3. Association between quality of life and pain characteristics. File 3; Figure S1. Flowchart outlining the inclusion of participants for this study.
