# Supplementary material for: Pain Characteristics and Quality of Life in Older People at High Risk of Future Hospitalization
Source: Int J Environ Res Public Health. 2021 Jan 22;18(3):958. doi: 10.3390/ijerph18030958 (PMC7908626; doi:10.3390/ijerph18030958)

Pain Characteristics and Quality of Life in Older People at High Risk of Future Hospitalization

Supplemental material File 1. Physical activity measures and multiple imputation statistical method.

Physical activity

Question 1: Respondents were asked about everyday physical activity during the last 6 months, such as walking or bicycling to the store, going out with dogs, gardening, shoveling snow or some other activity likewise (A =no everyday activity, B =activity at some time during the week, C =activity several times a week, D =almost daily or daily activity). Question 2: Respondents were also asked about exercise /sport /open-air activity (not included in the everyday life activities above). The answer alternatives were A) no activity, B) very little activity, C) mild form (soft) of activities such as walking at least once per week, D) more strenuous activities such as jogging, swimming, or gymnastics at least once per week, E) hard activities which demand great physical exertion and with regularity. A combination of the answers to both questions generated the physical activity level 1-4 (table 1).[1,2]

Multiple imputation

Missing data are often categorized into the following three types: missing completely at random (MCAR), missing at random (MAR), and missing not at random (MNAR).[3] Traditional statistical analysis such as linear regression is based on the assumption of MCAR. However, most clinical epidemiological research is neither MCAR nor MNAR but MAR.[4] Multiple imputation (MI) can be used to handle missing data under the MAR assumption, with a purpose of providing unbiased and valid estimates of associations based on information from the available data.[3,5] As described by Harel et al, MI consisted of 3 steps: data imputation (i.e. multiple imputation by chained equations (MICE)), analysis of each imputed data set (i.e. linear regression) and a final estimate of coefficients in the combination of all the imputed data sets (pooled).[5]
Before multiple imputation, we performed the analysis of missing data patterns. Table 2 showed number of missing cases in each variable of interest. A total of 1390 values (8.4%) were missing and 581 (70.4%) of the 825 cases contained at least one missing value.
Multiple imputation was applied using m =100 (iterations =50) imputed data set with predictive mean matching method and included all the variables of interest (predictors and outcomes). Several arguments and suggestions on the number of imputations have been discussed previously.[4,6,7] Using the pooled sample, we found that our models did not in fact change much as a result of imputing missing data. As shown in table 3, there were similar significant associations of all the domains of QoL with pain characteristics in comparison to the original data. However, the estimates' standard errors were slightly lower when missing cases were considered in the regression models.

References:

1. Peolsson A, Almkvist C, Dahlberg C, Lindqvist S, Pettersson S. Age- and sex-specific reference values of a test of neck muscle endurance. *Journal of manipulative and physiological therapeutics.* 2007;30(3):171-177.
2. Leijon ME, Stark-Ekman D, Nilsen P, et al. Is there a demand for physical activity interventions provided by the health care sector? Findings from a population survey. *BMC public health.* 2010;10:34-34.
3. Rubin DB. Inference and missing data. *Biometrika.* 1976;63(3):581-592.
4. Pedersen AB, Mikkelsen EM, Cronin-Fenton D, et al. Missing data and multiple imputation in clinical epidemiological research. *Clin Epidemiol.* 2017;9:157-166.

5. Harel O, Mitchell EM, Perkins NJ, et al. Multiple Imputation for Incomplete Data in Epidemiologic Studies. *American Journal of Epidemiology.* 2017;187(3):576-584.
6. Stuart EA, Azur M, Frangakis C, Leaf P. Multiple imputation with large data sets: a case study of the Children's Mental Health Initiative. *American journal of epidemiology.* 2009;169(9):1133-1139.
7. Heymans MWE, Iris. Applied Missing Data analysis Book with SPSS and (R) studio. In: Amsterdam: Heymans and Eekhout; 2019: https://bookdown.org/mwheymans/bookmi/.

Pain Characteristics and Quality of Life in Older People at High Risk of Future Hospitalization

Supplementary File 2. Table S1-S3.

Supplementary Table S1. Physical activity level generated by two questions.

Question 1 / Question 2	A	B	C	D
**A**	1	1	2	3
**B**	1	2	2	3
**C**	2	2	2	3
**D**	3	3	3	4
**E**	4	4	4	4

Supplementary Table S2. Missing cases in each variable.

	Missing N (%)	Valid N
**Pain duration**	263 (31.9)	562
**Physical activity level**	246 (29.8)	579
**SWLS scales**	168 (20.4)	657
**VAS Pain-7d**	121 (14.7)	704
**Frequency of feeling lonely**	105 (12.7)	720
**Frequency of feeling worried**	100 (12.1)	725
**PGCMS-subscales**	80 (9.7)	745
**EQ-5D index**	70 (8.5)	755
**Number of close relationships**	59 (7.2)	766
**Pain frequency**	43 (5.2)	782
**Education level**	28 (3.4)	797
**Number of used assistive technology**	24 (2.9)	801
**RAND-36$_{PF}$**	23 (2.8)	802
**Having relatives nearby**	22 (2.7)	803
**ADL-staircase**	18 (2.2)	807
**Living situations**	11 (1.3)	814
**Housing**	9 (1.1)	816

SWLS: Satisfaction with life scale; VAS: visual analog scale; PGCMS: Philadelphia Geriatric Center Morale Scale; PF: physical functioning; ADL: Activity of Daily Living.

Supplementary Table S3. Association between Quality of Life and pain characteristics.

DV / IV	EQ-5D index	Rand-36$_{PF}$	PGCMS-subscale	SWLS
**VAS Pain-7d**	-.002 (.0004) [c]	-.09(.04) [a]	-.003 (.002)	0.001 (0.01)
**Pain frequency**	-.05 (.01) [c]	-1.58 (.94) [a]	-.005 (.06)	-0.58 (0.21) [b]
**Pain extent**	-.005 (.001) [c]	-.07 (0.11)	-.02 (0.01) [b]	.02 (.02)
**Pain duration**	-.0004 (.0003)	-.04 (.03)	.003 (.002)	.003 (.007)

[a] p<.05, [b] p<.01, [c] p<.001. SWLS: Satisfaction with life scale; VAS: visual analog scale; PGCMS: Philadelphia Geriatric Center Morale Scale; PF: physical functioning.

Coefficients (standard error). IV: independent variables, DV dependent variables. Adjusted for sociodemographic (age, gender, education level, living situation and housing), functional status (number of used assistive technology, physical activity level, ADL-staircase) and social network (relatives living closely, number of important persons, frequency of feeling lonely and worried).

Supplementary File 3. Figure S1.

Fig 1. Flow chart outline the inclusion of participants for this study.

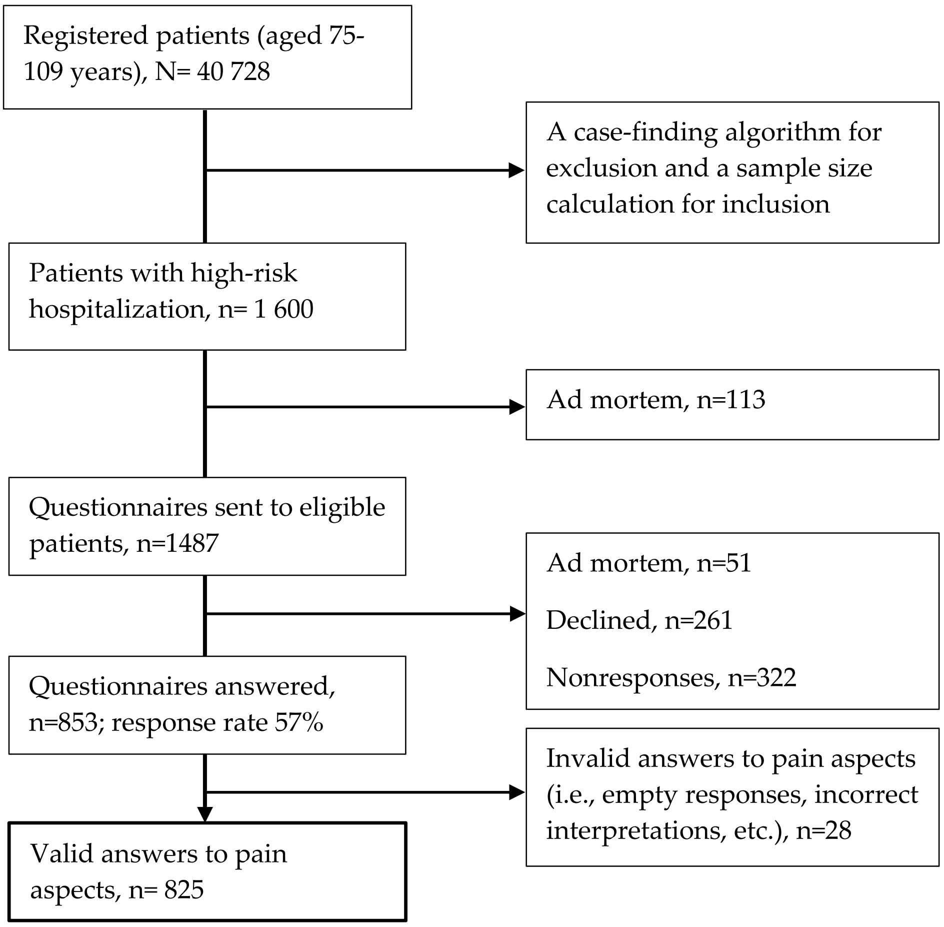

Supplement: Supplementary file 1 [file ijerph-18-00958-s001.pdf]
